# Behavioral interventions for individuals with fetal alcohol spectrum disorder: A review of systematic reviews

**DOI:** 10.1111/acer.70129

**Published:** 2025-08-11

**Authors:** Eric Flake, Elizabeth H. Lee, Kyle Patrick Apilado, Rachel Sayko Adams, Alyssa MacMahon, Tracey Perez Koehlmoos

**Affiliations:** ^1^ Center for Health Services Research Uniformed Services University of the Health Sciences Bethesda Maryland USA; ^2^ The Henry M. Jackson Foundation for the Advancement of Military Medicine, Inc. Bethesda Maryland USA; ^3^ Department of Pediatrics Uniformed Services University of the Health Sciences Bethesda Maryland USA; ^4^ Department of Health Law, Policy & Management Boston University School of Public Health Boston Massachusetts USA

**Keywords:** behavioral intervention, fetal alcohol spectrum disorder, review

## Abstract

**Background:**

Individuals diagnosed under the spectrum of fetal alcohol spectrum disorders (FASD) experience numerous cognitive and behavioral impairments, including learning disabilities, executive functioning dysfunction, and difficulties in emotional regulation. Studies of direct child‐centered nonpharmacological behavioral interventions to improve outcomes have steadily developed over the past few decades. Systematic reviews of this literature have documented the wide collection of studies, and an overall review of these reviews permits a single, comprehensive analysis encompassing behavioral intervention research.

**Methods:**

Electronic databases were searched for systematic reviews from 2005 to 2024. Included reviews reported the effectiveness of child‐centered behavioral, nonpharmacological interventions in noneducational settings for individuals with FASD from birth until the age of 18. Abstract screening, full‐text screening, and data extraction were conducted using Covidence. AMSTAR‐2 was utilized to assess the methodological quality of systematic reviews.

**Results:**

A total of seven systematic reviews were included for comprehensive analysis. Two reviews were of high quality, two were of low quality, and three were of critically low quality, as per AMSTAR‐2 grading criteria. Categories of behavioral interventions within systematic reviews included executive functioning interventions, self‐management interventions, social skill interventions, family‐based interventions, cognitive behavioral interventions, and applied behavior analysis‐based treatment. While numerous positive outcomes were identified across several behavioral interventions, the systematic reviews identified multiple limitations, such as high risk of bias and small sample sizes.

**Conclusion:**

Numerous positive outcomes were identified from among systematic reviews regarding FASD interventions; however, the current evidence base is limited by methodological weaknesses and potential risks of bias. Further research and implementation are necessary to strengthen the delivery of interventions and continue improving outcomes for individuals with FASD.

## Graphical abstract

This review assessed multiple systematic reviews regarding behavioral interventions for individuals with fetal alcohol spectrum disorders (FASD). Multiple positive outcomes were identified from studied FASD interventions, particularly toward executive functioning, social problem‐solving skills, and emotional regulation. However, the current evidence base is limited by methodological weaknesses and potential risks of bias. Future research should prioritize rigorous study designs, standardized outcome measures, and diversification of targeted populations to increase the strength of implementation.

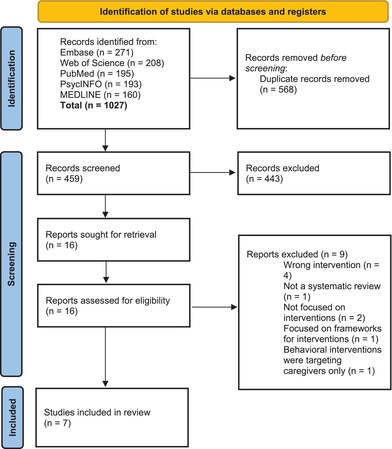

## BACKGROUND

The collection of work by Paul Lemoine, Elizabeth Turner, and Christy Ulleland has long established that in‐utero alcohol exposure is harmful to fetal development (Jones et al., 1973; Jones & Smith, 1973; Lemoine et al., 2003; Turner, 1979). This exposure can lead to a range of lifelong physical and neurodevelopmental disabilities, falling under the umbrella of fetal alcohol spectrum disorders (FASD). The spectrum of FASD diagnoses includes fetal alcohol syndrome (FAS), partial fetal alcohol syndrome (PFAS), alcohol‐related neurodevelopmental disorder (ARND), alcohol‐related birth defects (ARBD), and neurobehavioral disorder associated with prenatal alcohol exposure (ND‐PAE) (Bertrand et al., 2005; Hagan et al., 2016). There are more than five different diagnostic criteria worldwide, some of which are not recognized in the United States. These include the Canadian guidelines, Australian diagnosis guidelines, and the National Institute for Health and Care Excellence guidelines in the United Kingdom (Cook et al., 2016; Bower & Elliott, 2016; National Institute for Health and Care Excellence, 2022). Decades of research on the lifelong effects of prenatal alcohol exposure have shown increased risk for impairments in cognitive, behavioral, and physical health, as well as mental health disorders and substance misuse (Denny et al., 2017; Mattson et al., 2011; Temple et al., 2021). People with FASD may be impacted by a range of brain and body concerns, including intellectual and learning disabilities, adaptive and executive dysfunction, emotional and behavioral difficulties, attention deficits and hyperactivity, speech and language delays, motor deficits, and impairments in visual–spatial reasoning (Himmelreich et al., 2020; Mattson et al., 2019).

Several studies have identified the public health burden of FASD. A 2018 case ascertainment study provided a conservative estimate of up to 5% of first‐grade students meeting FASD diagnostic criteria in the United States (May et al., 2018). This brain‐based neurodevelopmental condition that affects the entire body requires medically necessary interventions to assist with self‐regulation and skill development for activities of daily living. Conservative estimates of the annual cost of care for all individuals living with FASD range from $1.29 billion to $10.1 billion (Greenmyer et al., 2020).

The widespread impact of FASD on children and their caregivers across the lifespan has motivated studies of potential behavioral interventions to improve outcomes for people with FASD. While there were several intervention studies published between the 1990s and 2000s, they were mainly focused on administering psychostimulant medications in children with co‐occurring FASD and ADHD (Paley & O'Connor, 2009). Following the Children's Health Act in 2000, the US Centers for Disease Control and Prevention issued a call to action, offering funding to researchers to develop and scientifically evaluate behavioral interventions for children with FASD (Olson et al., in Bertrand, 2009). The call to action resulted in a few of the first FASD‐specific interventions covering a range of needs, from the Children's Friendship Training (CFT) for knowledge of appropriate social behavior (O'Connor et al., 2006), to the Math Interactive Learning Experience program for improving mathematical ability and behavior management (Coles et al., 2009; Kable et al., 2007), the neurocognitive habilitation program for enhancing executive functioning and self‐regulation skills (Wells et al., 2012), and Families Moving Forward (FMF), directed at family members of children with FASD, for family behavioral consultations and support (Olson et al., in Bertrand, 2009).

Following the increase in FASD behavioral intervention studies over the past 18 years, several researchers have documented the available evidence base through systematic reviews (Flannigan et al., 2020; Hilly et al., 2023; Ordenewitz et al., 2021; Peadon et al., 2009; Premji et al., 2007; Reid et al., 2015). Individualized interventions for children with FASD are necessary because their needs are shaped by layered developmental challenges and often compounded by socioeconomic factors, trauma histories, and disrupted caregiving. These complexities make one‐size‐fits‐all approaches ineffective, and personalized interventions are essential to support meaningful growth, improve function, and promote long‐term well‐being. Building firm evidence for child‐centered FASD behavioral interventions helps ensure that treatment options have been validated in methodologically sound studies, allowing clinical practitioners to make better‐informed decisions and enabling medical systems to recognize best practices.

The goal of this review of reviews was to provide a comprehensive synthesis of systematic, review‐level evidence on child‐centered, nonpharmacological, clinically based behavioral interventions directed at individuals with FASD, drawing meaningful conclusions for clinical practice. Specifically, this review aims to: collate and appraise the evidence from recent systematic reviews on behavioral interventions for FASD; utilize systematic reviews to describe the nature and reported strength of the recommended interventions for people with FASD from systematic reviews; and identify high‐quality evidence from the peer‐reviewed literature to inform clinical practice. This review distinguishes child‐centered therapy from parent‐focused therapy, emphasizing interventions specifically designed to support children and adolescents directly.

## METHODS

A review of reviews was conducted according to the recommendations of Smith et al., and the articles were assessed by their alignment with the general framework of Preferred Reporting Items for Systematic Reviews and Meta‐Analyses (PRISMA) (Liberati et al., 2009; Page et al., 2021; Smith et al., 2011). A review of reviews methodology examines the available research on a subject through a rigorous assessment of existing systematic reviews (Aromataris et al., 2015). This framework makes it possible to appraise the quality of systematic reviews, compare and contrast their findings, and evaluate the strength of their conclusions (Smith et al., 2011). This is an essential source of information that highlights the best quality reviews on the topic and creates a strong evidence base for clinical practice (Smith et al., 2011). This study was not registered. A protocol was not prepared for this study.

### Eligibility criteria

Articles were considered for inclusion if they: (1) were a systematic review (as defined below); (2) reported on the effectiveness of child‐centered nonpharmacological, behavioral interventions for FASD that were performed in noneducational settings for children (birth to 18 years) or their family unit; and (3) were published in English, from 2005 to 2024. The study date range was selected due to the introduction of novel, child‐centered therapies for FASD. Although the search process did not strictly exclude non‐English‐based literature, English articles were still primarily included due to translational capacity. Reviews were considered systematic if they: (1) included clearly stated objectives with reproducible methodology; (2) provided a systematic search strategy in alignment with eligibility criteria; (3) presented and synthesized the findings of the included studies. We excluded systematic reviews that did not meet the three aforementioned criteria or only presented a narrative form of literature review.

For the purpose of this review, behavioral interventions were defined as “treatment strategies that emphasize functional behavior‐environment relationships” (Coyne & Gross, 2001). The main goal of these interventions is “to enhance adaptive behaviors and eliminate or reduce maladaptive behaviors in daily life” (Coyne & Gross, 2001). Included interventions were those delivered directly to the individual living with FASD, excluding those that targeted the caregiver exclusively. Examples of child‐centered behavior interventions included behavior skills training, self‐management and regulation programs, applied behavioral analysis, social skills and communication training, and child‐caregiver dyad behavior intervention programs (e.g., parent–child interaction therapy). This specified behavioral focus aims to highlight a medical model where children are referred to therapeutic services for identified deficits. These interventions can be clearly delineated as medically necessary to address the functional limitations impacting the child's health and development.

### Search strategy and study selection

To identify systematic reviews, a combination of biomedical and psychological databases was searched. The databases accessed included PubMed, Embase, APA PsycInfo, Web of Science, and Medline. The following framework of keywords was used to build the literature search in each database: terms related to FASD, infants, children, adolescents, and behavioral interventions. The complete search strategy used for each database is detailed in Data S1.

Two reviewers (ENL and EF) screened the titles and abstracts of each article in Covidence, and a third reviewer (EHL) resolved any conflicts. Next, two reviewers (ENL and EF) obtained each potentially eligible article for full‐text screening. Any disagreements regarding the final inclusion of a study were resolved by discussion with the study team.

### Data extraction

One reviewer (ENL) conducted data extraction for the included systematic reviews. From each included systematic review, the following information was extracted: authors, type of review, number of relevant primary studies, years of relevant studies, included study design(s), description of behavioral interventions, population characteristics (country, sample size, age range, and diagnoses), intervention setting, and key findings.

### Quality assessment

The methodological quality of the included systematic reviews was assessed by an author using the AMSTAR‐2 (A Measurement Tool to Assess Systematic Reviews). AMSTAR‐2 categorizes reviews based on critical and noncritical weaknesses across its 16 domains. A review is rated high quality if it has no or only one noncritical weakness, low quality if it has more than one critical flaw, and critically low quality if it contains more than one critical flaw. AMSTAR‐2 was chosen for its ability to evaluate reviews of both randomized and nonrandomized studies and its content validity and good inter‐rater agreement (Shea et al., 2017).

## RESULTS

### Systematic reviews

**FIGURE 1:**
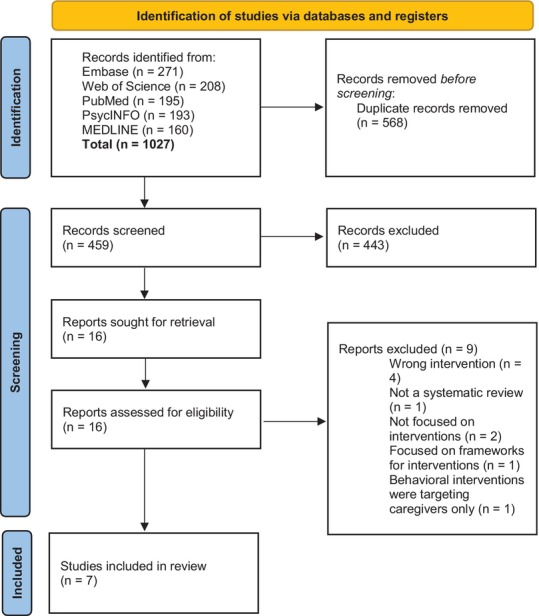
PRISMA flowchart of abstract screening process. *Source*: Page et al. (2021). This work is licensed under CC BY 4.0. To view a copy of this license, visit https://creativecommons.org/licenses/by/4.0/.

Seven systematic reviews met inclusion criteria after screening, as depicted in Figure 1 (PRISMA flowchart) and Table 1. Two of these included meta‐analyses. The number of primary studies included within the reviews ranged from 5 to 33. After examining the included primary studies, the number of relevant behavioral intervention studies that could be assessed ranged from 1 to 15 (Data S2). Only one primary study was unique to one systematic review, with all other studies being referenced by two or more reviews (Table 2). A total of nine reviews were examined in full‐text review but were subsequently excluded after screening. While these excluded reviews provided additional insight into treatment for FASD, they did not meet this study's inclusion criteria and fell outside its objectives of highlighting clinical child‐centered interventions (Data S3).

**TABLE 1:** Included systematic reviews.

Title	Source	Year
Systematic review of interventions for children with fetal alcohol spectrum disorders	Peadon et al.	2009
Systematic review of fetal alcohol spectrum disorder interventions across the life span	Reid et al.	2015
A systematic review of interventions to improve mental health and substance use outcomes for individuals with prenatal alcohol exposure and fetal alcohol spectrum disorder	Flannigan et al.	2020
Evidence‐based interventions for children and adolescents with fetal alcohol spectrum disorders: A systematic review	Ordenewitz et al.	2021
Effectiveness of interventions for school‐aged‐children and adolescents with fetal alcohol spectrum disorder: A systematic review and meta‐analysis	Hilly et al.	2023
Interventions for improving executive functions in children with fetal alcohol spectrum disorder (FASD): A systematic review	Betts et al.	2022
A tornado in the family: Fetal alcohol spectrum disorder and aggression during childhood and adolescence: A scoping review	Champagne et al.	2023

**TABLE 2:** Systematic review references to primary behavioral intervention studies.

Intervention	Publications (title, author)	Number of references by systematic reviews^a^	Systematic review references
Alert Program for Self‐Regulation	Improving executive functioning in children with fetal alcohol spectrum disorders using the alert program for self‐regulation (Ph.D. thesis) (Nash et al., 2015) Improving executive functioning in children with fetal alcohol spectrum disorders (Nash et al., 2015) Self‐regulation therapy increases frontal gray matter in children with fetal alcohol spectrum disorder: evaluation by voxel‐based morphometry (Soh et al., 2015)	6	Betts et al. (2022) Champagne et al. (2023) Flannigan et al. (2020) Hilly et al. (2023) Ordenewitz et al. (2021) Reid et al. (2015)
Children's Friendship Training (CFT)	A controlled social skills training for children with fetal alcohol spectrum disorders (O'Connor et al., 2006) Impact of a social skills intervention on the hostile attributions of children with prenatal alcohol exposure (Keil et al., 2010) Translation of an evidence‐based social skills intervention for children with prenatal alcohol exposure in a community mental health setting (O'Connor et al., 2006)	5	Flannigan et al. (2020) Hilly et al. (2023) Ordenewitz et al. (2021) Peadon et al. (2009) Reid et al. (2015)
Families on Track (FOT)	Findings from the families on track intervention pilot trial for children with fetal alcohol spectrum disorders and their families (Petrenko et al., 2017) Six‐month follow‐up of the families on track intervention pilot trial for children with fetal alcohol spectrum disorders and their families (Petrenko et al., 2019)	4	Betts et al. (2022) Champagne et al. (2023) Flannigan et al. (2020) Ordenewitz et al. (2021)
GoFAR	A metacognitive strategy for reducing disruptive behavior in children with fetal alcohol spectrum disorders: GoFAR pilot (Coles et al., 2015) Improving FASD children's self‐regulation: Piloting phase 1 of the GoFAR intervention (Kable et al., 2007) GoFAR: improving attention, behavior and adaptive functioning in children with fetal alcohol spectrum disorders: brief report (Coles et al., 2018)	5	Betts et al. (2022) Champagne et al. (2023) Flannigan et al. (2020) Hilly et al. (2023) Ordenewitz et al. (2021)
Neurocognitive Habilitation Therapy	Neurocognitive habilitation therapy for children with fetal alcohol spectrum disorders: An adaptation of the Alert Program (Wells et al., 2012)	5	Betts et al. (2022) Flannigan et al. (2020) Hilly et al. (2023) Ordenewitz et al. (2021) Reid et al. (2015)
Parent–Child Interaction Therapy (PCIT)	Parent–child interaction therapy: Application of an evidence‐based treatment to reduce behavior problems among children with fetal alcohol spectrum disorders (Gurwitch et al., reported in Bertrand, 2009)	2	Flannigan et al. (2020) Reid et al. (2015)
Parents Under Pressure (PuP)	Feasibility study of a family‐focused intervention to improve outcomes for children with FASD (Reid et al., 2015)	2	Betts et al. (2022) Flannigan et al. (2020)
Self‐Management Intervention	Effects of a self‐management intervention to improve behaviors of a child with fetal alcohol spectrum disorder (Griffin & Copeland, 2018) Teaching self‐management strategies to a child with fetal alcohol spectrum disorder to increase independent task completion within typical home routines (Copeland et al., 2021)	2	Flannigan et al. (2020) Hilly et al. (2023)
Verbal Behavior Intervention: ABA‐Based Therapy	The clinical application of applied behavior analysis in a child with partial fetal alcohol syndrome: A case study (Connolly et al., 2016)	1	Flannigan et al. (2020)

^a^ Number of systematic reviews citing the intervention.

### Quality assessment

The quality assessment ratings of each review are presented in Table 3. Among the seven reviews, two were determined to be of high quality, two were of low quality, and three were critically low. Both high‐quality reviews performed a meta‐analysis using appropriate statistical methods to synthesize results and assessed the potential for publication bias among studies. All reviews clearly outlined the inclusion criteria, and potential conflicts of interest were addressed. However, there were often variations in critical items, such as prior establishment of review methods, using proper techniques for assessing risk of bias, and justifying reasons for excluding studies, leading to lower overall scores among the five lower quality reviews.

**TABLE 3:** AMSTAR‐2 critical appraisal.

		Betts et al. (2022)	Champagne et al. (2023)	Flannigan et al. (2020)	Hilly et al. (2023)	Ordenewitz et al. (2021)	Peadon et al. (2009)	Reid et al. (2015)
1	Did the research questions and inclusion criteria for the review include the components of PICO?	Yes	Yes	Yes	Yes	Yes	Yes	Yes
2^a^	Did the report of the review contain an explicit statement that the review methods were established prior to the conduct of the review and did the report justify any significant deviations from the protocol?	Yes	No	Partial yes	Yes	No	No	Partial yes
3	Did the review authors explain their selection of the study designs for inclusion in the review?	Yes	No	Yes	Yes	No	Yes	No
4^a^	Did the review authors use a comprehensive literature search strategy?	Yes	Partial yes	Partial yes	Yes	Partial yes	Partial yes	Yes
5	Did the review authors perform study selection in duplicate?	Yes	Yes	Yes	Yes	Yes	Yes	Yes
6	Did the review authors perform data extraction in duplicate?	Yes	Yes	No	Yes	Yes	Yes	No
7^a^	Did the review authors provide a list of excluded studies and justify the exclusions?	Yes	No	No	Yes	No	No	No
8	Did the review authors describe the included studies in adequate detail?	Yes	Yes	Yes	Yes	Partial yes	Yes	Partial yes
9^a^	Did the review authors use a satisfactory technique for assessing the risk of bias (RoB) in individual studies that were included in the review?	Yes	No	Partial yes	Yes	No	Partial yes	Partial yes
10	Did the review authors report on the sources of funding for the studies included in the review?	Yes	No	No	Yes	No	No	No
11^a^	If meta‐analysis was performed did the review authors use appropriate methods for statistical combination of results?	Yes	Not applicable	Not applicable	Yes	Not applicable	Not applicable	Not applicable
12	If meta‐analysis was performed, did the review authors assess the potential impact of RoB in individual studies on the results of the meta‐analysis or other evidence synthesis?	Yes	Not applicable	Not applicable	Yes	Not applicable	Not applicable	Not applicable
13^a^	Did the review authors account for RoB in individual studies when interpreting/discussing the results of the review?	Yes	No	Yes	Yes	No	Yes	Yes
14	Did the review authors provide a satisfactory explanation for, and discussion of, any heterogeneity observed in the results of the review?	Yes	No	No	Yes	No	Yes	Yes
15^a^	If they performed quantitative synthesis did the review authors carry out an adequate investigation of publication bias (small study bias) and discuss its likely impact on the results of the review?	Yes	Not applicable	Not applicable	Yes	Not applicable	Not applicable	Not applicable
16	Did the review authors report any potential sources of conflict of interest, including any funding they received for conducting the review?	Yes	Yes	Yes	Yes	Yes	Yes	Yes
Overall quality of review^b^		High	Critically low	Low	High	Critically low	Critically low	Low

^a^ Critical item.

^b^ Quality rating: high: No or one noncritical weakness; moderate: More than one noncritical weakness; low: One critical flaw with or without non‐critical weaknesses; critically low: More than one critical flaw with or without noncritical weaknesses.

### Characteristics of reviews

Across the systematic reviews, the years of relevant behavioral intervention studies ranged from 2006 to 2021. The majority of reviews included a combination of randomized control trials, case–control trials, and single‐case experimental designs, or case studies. The included primary studies were most often conducted in the United States or Canada, with one study in Australia. Sample sizes ranged from 85 to 460 children, aged 3–12 years. The most common diagnoses among children in the included studies were FAS, pFAS, and ARND, with only one study targeting a child with ND‐PAE. Locations in included studies primarily consisted of clinical settings, home settings, community centers, and a few unspecified therapeutic settings.

### Review focus areas

Although we limited our data extraction within the reviews to studies addressing behavior that met our established child‐centered intervention criteria, the reviews often included other varieties of interventions for FASD. Champagne et al. limited their focus to behavioral interventions, specifically targeting aggressive behavior in children and adolescents with FASD (2023). Four reviews assessed all varieties of interventions for children with FASD. Reid et al. (2015) observed interventions across the lifespan, while three other reviews examined interventions for children and adolescents (Hilly et al., 2023; Ordenewitz et al., 2021; Peadon et al., 2009). Apart from individual, direct behavioral interventions, these reviews identified and evaluated interventions in attention, education, language/speech, memory skills, and fine motor functions. Betts et al. (2022) focused on interventions that may improve either indirect or direct measures of executive functioning in children. Flannigan et al. concentrated on interventions across the lifespan that may benefit mental health and substance use outcomes for individuals with FASD.

Outcome effects were also reported in two of the collected systematic reviews. Hilly et al. observed a small but significant pooled effect size (*g* = 0.29; 95% CI 0.15, 0.43), with statistically significant treatment effects for overall behavioral outcomes (*g* = 0.21; 95% CI 0.10, 0.33), as well as overall activity outcomes (*g* = 0.36; 95% CI 0.17, 0.54). Specifically, among outcomes with multiple reports, emotional regulation (*g* = 0.43; 95% CI 0.16, 0.69), social cognition (*g* = 0.30; 95% CI 0.01–0.59), and externalizing behaviors (*g* = 0.26; 95% CI 0.03, 0.50) experienced statistical significance. Betts et al. assessed outcome effects for eligible studies. Despite positive effects, overall composite measures observed no statistically significant treatment effects across global executive functioning (SMD = 0.21; 95% CI = −0.40, 0.82), behavioral regulation (SMD = 0.18; 95% CI −0.42, −0.79), and metacognition (SMD = 0.23; 95% CI −0.72, 1.19).

### Categories of behavioral interventions

*Executive functioning interventions* were evaluated across six reviews, with studies focusing upon the regulation of emotions and behaviors in response to external stimuli. Reviewed studies primarily utilized the Alert intervention, along with Neurocognitive Habilitation Therapy. Studies consisted of male and female children aged 6–11 years, with program durations ranging between 12 and 14 weeks, meeting once a week. All reviews found positive benefits when directly measuring outcomes in children, with studies displaying slight improvements in executive functioning, emotional regulation, and social problem‐solving skills (Nash et al., 2015; Soh et al., 2015). Reviews also found that adaptations of the Alert program created significant development of gray matter in participants (Flannigan et al., 2020; Ordenewitz et al., 2021; Reid et al., 2015). Perceived outcomes were also recorded through standardized questionnaires. Despite the positive outcomes observed directly in children, caregivers reported no perceived improvement in executive functioning. A meta‐analysis conducted by Betts et al. (2022) found that overall developments in behavioral regulation were generally statistically nonsignificant.

*Self‐management interventions* pertaining to task completion and establishing independence at home were investigated among two reviews. Included articles were single‐case experimental studies of a 9‐year‐old boy and an 11‐year‐old boy that lasted 14–20 weeks and required individual subjects to complete tasks in a household setting. Reviews observed a reduction in both prevalence and intensity of behavioral issues, including a decrease in argumentativeness (Copeland et al., 2021; Griffin & Copeland, 2018). Included reviews noted concerns about the high risk of bias within these studies due to sampling issues, which undermine the reliability of this intervention type (Flannigan et al., 2020; Hilly et al., 2023).

*Social skill interventions* targeting the development of social interaction for children with FASD were evaluated in five reviews. The primary program of study was the Children's Friendship Training, lasting 12 weeks and composed of male and female children aged 6–12 years. Reviews observed favorable outcomes for this intervention in children with FASD, with results indicating improvements in social interaction with peers, along with a reduced tendency to perceive external behaviors as hostile (Keil et al., 2010; O'Connor et al., 2006). Parents continued to document improved social skills and behaviors, with overall evidence suggesting that social skill interventions, such as Children's Friendship Training, may be effective in social skill development regardless of PAE diagnosis (Flannigan et al., 2020). However, two reviews found potential risks of bias within a significant study of this intervention, and a pooled meta‐analysis of social skills outcomes was determined to be statistically nonsignificant (Hilly et al., 2023; Peadon et al., 2009).

*Parent‐child dyad and family‐focused child‐centered interventions* were evaluated across five reviews, consisting of numerous programs synchronously targeting both the parent/caretaker and child behaviors to promote healthier family environments and interactions. The intensity and directness of child‐centered interactions within these interventions potentially vary, as parents are often the primary source of instruction. Programs, such as Families on Track and Parents Under Pressure, as well as Parent–Child Interaction Therapy (PCIT), lasted 14–30 weeks, involving male and female children aged 3–12 years. Reviews reported mixed results across the discrete interventions. Reviews including Families on Track reported positive outcomes, such as enhanced caregiver knowledge and understanding of FASD, as well as slight reductions in negative behaviors (Champagne et al., 2023; Flannigan et al., 2020). Despite the positive outcomes, these reviews also noted diminishing long‐term returns, with Flannigan et al. (2020) reporting a decline in emotional regulation and even a regression in self‐esteem after long‐term administration of the intervention.

*Parents under pressure*, which focuses on the parent but is delivered with the child present, had mixed outcomes. Despite general improvements in family functioning, feelings of support, and an overall reduction in child distress, only one participant displayed statistically significant positive benefits in psychosocial outcomes (Flannigan et al., 2020). Betts et al. (2022) further raised criticism surrounding the single‐case nature and high risk of bias within the Parents Under Pressure Program, questioning the overall statistical significance of findings. Reviews documented improvements in child behavior problems and caregiver stress for Parent–Child Interaction Therapy (PCIT) sessions (Flannigan et al., 2020; Reid et al., 2015). However, Flannigan et al. found these results to be nonsignificant compared with the control group.

Behavior regulation interventions, such as the GoFAR program, aim to improve metacognition and behavioral regulation skills among children with FASD (Coles et al., 2015). Consensus among multiple reviews was that cognitive behavioral interventions were impactful among children with FASD, revealing improvements in attention and adaptive functioning, as well as reductions in disruptive behaviors. Reviews also established that parental and caregiver engagement supported positive behaviors throughout the intervention (Flannigan et al., 2020; Kable et al., 2007; Ordenewitz et al., 2021). Yet, a meta‐analysis by Betts et al. (2022) found that although positive, the results of a major study of this intervention were not statistically significant for outcomes, such as metacognition. Meta‐analysis results from Hilly et al. (2023) found similarly mixed results for GoFAR, as outcomes on externalizing behaviors were slightly statistically significantly positive, while outcomes on internalizing behaviors were nonsignificant.

Applied behavioral analysis‐based treatment was reviewed by Flannigan et al., consisting of a one‐to‐one intervention case study of a 3‐year‐old girl. Lasting 23 months for 15 hours a week, researchers and caregivers reported significant behavioral and clinical improvements, particularly in functional communication and adaptive behavior (Flannigan et al., 2020). However, critical appraisal of the study determined a weak rating, especially regarding study design, confounding variables, and selection bias.

## DISCUSSION

Of the seven systematic reviews identifying categories for the specific inclusion of child‐centered therapy, two were determined to be of high quality, two were of low quality, and three were of critically low quality. Among these, we identified a diverse set of behavioral interventions and strategies that can help improve outcomes and alleviate the challenges associated with FASD. These reviews not only highlight the efficacy of such interventions but also the limitations and methodological gaps that may limit the broad implementation of these interventions. While analysis of interventions was extensive, this review emphasizes the need to not only conduct further assessments of child‐centered interventions but also to improve the quality of future systematic reviews. An additional highlight is the limited studies that focus on child‐centered treatment for FASD, compared with treatment for other children with neurodevelopmental disorders, such as Autism or ADHD.

### Review quality

While reviews of high or moderate quality provided a good assessment of behavioral interventions, the majority of reviews were of low or critically low quality. This mixed overall quality of reviews limits the confidence of general conclusions, with a key concern being the lack of established methodology for low‐quality reviews. The potential bias and reduced validity of results underscore the need for a consistent standard of future reviews to adequately assess behavioral intervention outcomes with improved confidence.

### Type of intervention

It has been well established that, depending on the severity, FASD can have lifelong and even irreversible impacts on neurological development and overall functioning (Temple et al., 2021). Given FASD's significant impact upon the brain's prefrontal cortex, impaired executive functioning is widely recognized as a contributor to social and behavioral challenges observed in individuals living with FASD (Betts et al., 2022; Khoury et al., 2015). Emerging neuroplasticity research highlights that interventions prioritizing executive functioning can support improved skills and development. Programs, such as Alert, have not only been extensively implemented and explored but have also demonstrated promise in improving self‐regulation, shifting away from the dispelled historical perception that FASD establishes static encephalopathy or irreversible brain damage (TherapyWorks, Inc., 2019). Positive improvements in areas, such as emotional regulation and executive functioning with confirmatory brain development, are present, showing that certain behavioral and cognitive functions can improve with child‐centered support (Soh et al., 2015). However, the difficulty in measuring such outcomes, the ongoing discrepancy between observed outcomes and caregiver perceptions, and the variability of statistical findings call for additional studies.

Another hallmark challenge for children with FASD is self‐regulation and adaptive behavior. Independent interventions for the improvement of these skill sets demonstrate promising results with observed outcomes of reduced behavioral issues after consistent weekly sessions (Flannigan et al., 2020; Hilly et al., 2023). Such findings reinforce the idea that regular, structured intervention is impactful in achieving positive outcomes. However, the single‐case nature and the high risk of bias identified within reviews warrant cautious interpretation and make it challenging to determine the generalizability of outcomes to other demographic groups or in implementation environments. The use of self‐regulation interventions calls for developing a standardized, accessible methodology that may enable valid outcomes.

Delayed social skills are extensively perceived across numerous studies of neurodevelopmental disorders (Feller et al., 2024; Löytömäki et al., 2023). Deficits in social competence are highly prevalent among individuals with FASD and may contribute to challenges, such as academic delinquency and overall poor judgment (O'Connor et al., 2006). Reviews that assessed interventions to improve social interaction abilities demonstrated enhanced social skills and a reduced tendency to perceive peer behaviors as hostile. The lack of diversity challenges the availability, accessibility, and cultural adaptability of social interventions to other geographic regions and demographic populations. A lack of statistical significance may be attributed to sample size, noncomprehensive outcome measures, or even discrepancies in caregiver and practitioner observations. The absence of such significance does not necessarily indicate a deficient intervention, but may need refined outcome measures and continuing, rigorous assessments.

Parent‐Only, Parent–Child Dyad co‐treatment models, or Family‐Based treatment, which include the child, are among the most prevalent interventions for children with FASD. Many evidence‐based models focused primarily on parent training specifically, with the most notable parent training curriculum being Families Moving Forward. This strategy of improving family dynamics and the caregiver–child relationship by instructing caregivers on how to appropriately respond to behaviors has been extensively studied. It is likely the first line of intervention due to the feasibility of educating and supporting the caregiver instead of child‐centered therapy. This review's focus on child‐centered therapy is not intended to discount the value of caregiver‐focused interventions, but rather to acknowledge that the evidence for this intervention is already well established, and thus a child‐centered review was necessary. Integration of the child within the intervention at the same time as the caregiver, such as with Families on Track, showed improvements in family functioning and child distress, further highlighting how family context plays a critical role in intervention success. However, the diminishing returns indicate a plateau of benefits for the intervention (Flannigan et al., 2020). In turn, this plateau suggests that more focused, direct intervention may be necessary to further promote individual neurodevelopmental progress.

### Efficacy of intervention

Caregiver involvement was identified as a crucial factor in supporting positive behaviors throughout the intervention process for both cognitive behavior therapy and applied behavior analysis. Programs, such as GoFAR, report improved attention and better regulatory calming skills. Despite the positive outcomes, studies were hampered by a lack of statistical significance, potentially attributed to the lack of sample size (Betts et al., 2022). Further investigation and larger sample sizes are necessary to determine success for broader implementation. A single, intensive case study of applied behavioral analysis (ABA) showed significant improvements in functional communication and adaptive behaviors. However, the intensity and two‐year duration of implemented interventions raise questions surrounding feasibility and expansion.

### Opportunities and challenges

Across these intervention types, several overarching opportunities and challenges emerged. Studies implementing longer duration interventions demonstrated significant benefits among reviews, suggesting that modern interventions should continue to prioritize sustained support and engagement periods (Hilly et al., 2023). Comprehensive engagement of both child and caregiver is also key in maximizing benefits (Champagne et al., 2023; Flannigan et al., 2020). Limited sample size is a common challenge for studies, which hinders the statistical analysis of outcomes and raises questions about how studies approach different and broader population groups (Flannigan et al., 2020). Some studies also relied on caregiver perceptions, which may provide bias and impact the standards of outcome measures. Evidence of long‐term impact across an elongated time span was also limited, as reviews centered on children rather than across the lifespan; study interventions lacked participants over 12 years of age, overlooking potential opportunities and gaps for teenage individuals with FASD. This creates difficulty in determining whether the benefits persisted beyond the study's conclusion as well as adolescence. Longer duration studies of interventions are thus encouraged to help refine interventions and ensure lifelong benefits.

### Strengths and limitations

Strengths of this study include its design, which is a comprehensive synthesis of multiple systematic reviews capturing over 18 years of interventional data. This approach provides a broad perspective on behavioral interventions for children with FASD. Additionally, this consolidation of reviews identifies common, overarching themes and strategies, as well as gaps for further study concerning child‐centered therapies. The application of the AMSTAR‐2 critical appraisal tool allowed proper evaluation of the methodological quality of systematic reviews. Assessing key domains, such as study design and risk of bias, ensures a transparent and structured review of the quality and reliability of prior conclusions while identifying critical gaps for improvement. Together, these findings help pave the way for future research.

However, the interpretability and generalizability of this work are not without limitations. Differences in study population size and characteristics, intervention methods, and outcome measures may introduce bias and limit the interpretation and application of findings. Furthermore, the conclusions presented may be biased due to the AMSTAR‐2 quality of each systematic review. Many systematic reviews were rated low or critically low‐quality, limiting the overall reliability of synthesized results. Although our search process comprehensively encompassed sources well within our inclusion criteria, we did not utilize Cochrane for systematic review searches, which may reduce transparency and reproducibility for future searches. This review was also not registered, which may limit its transparency and reproducibility. Furthermore, limiting inclusion criteria to English language articles may have resulted in excluding non‐English language reviews and studies. There is a pressing need for improved outcome measures, consistent methodology, and bias mitigation within systematic reviews to validate overall results.

### Future directions

Numerous gaps in the current literature warrant further assessment. An exclusion of older adolescent study groups is a considerable gap in these research endeavors, especially as they face different, less‐supervised social environments. There is also limited exploration of interventions that may account for socioeconomic, cultural, and linguistic differences, as many interventions are English‐focused and developed in Western nations (Rockhold et al., 2024). Furthermore, only a small number of studies reported participants from multiple ethnic backgrounds, and even within these, minority groups were still limited, as study samples were predominantly composed of Caucasian participants. These findings were similarly observed by the review conducted by Champagne et al. (2023).

Building upon these needs, child‐centered therapeutic interventions for individuals with FASD are critical. Future research must implement methodological rigor, recruit larger and diverse sample sizes, and utilize standardized outcome measures. Long‐term outcomes should also be assessed to determine potential benefits across significant developmental periods.

Interventions must be adaptable and accessible, addressing socioeconomic barriers while integrating caregiver and, when possible, child perspectives. There is a critical need for child‐centered therapies that target self‐emotional regulation, executive functioning, and social skills, with expanded inclusion of adolescents and adults. Two‐generation, family‐centered models should also be further refined, ensuring sustained engagement and adaptive support. This research would complement the existing success of parent‐directed interventions for FASD. Additionally, these interventions can assist all individuals with complex neurodevelopmental challenges beyond just those with FASD. A focus on feasible, scalable, and low‐burden delivery models, such as telehealth or community‐based formats, is imperative to bridge access gaps and improve outcomes by delivering interventions within familiar environments (Skorka et al., 2020).

## CONCLUSION

This review of reviews yields several lessons learned over nearly two decades of research on behavioral interventions for individuals impacted by prenatal alcohol exposure. On the whole, reviews highlighted the effectiveness of providing consistent child‐centered support with caregiver engagement on behavior modification. Interventions often fostered improvements in emotional regulation, executive functioning, and social problem‐solving skills, highlighting areas of potential success for individuals with FASD. The challenges present in current evaluations of behavioral interventions, such as issues in sample sizing and bias mitigation, may be overcome by continued study and implementation of interventions.

## AUTHOR CONTRIBUTIONS

EF, EHL, and TPK designed the study. EF and EHL conducted screening, data extraction, and quality assessment of reviews. TPK provided funding for the project. All authors assisted in the drafting, revising, and approval of the final manuscript.

## FUNDING INFORMATION

This study was funded through the Defense Health Agency, Grant #HU00012320112.

## CONFLICT OF INTEREST STATEMENT

None.

## ETHICS STATEMENT

This review of systematic reviews included previously published reviews, so no patient information was included in the study.

## DISCLAIMER

The contents of this publication are the sole responsibility of the authors and do not necessarily reflect the views, assertions, opinions, or policies of the Uniformed Services University of the Health Sciences (USUHS), the Henry M. Jackson Foundation for the Advancement of Military Medicine, Inc. (HJF), the Department of Defense (DoD), or the Departments of the Army, Navy, or Air Force. Mention of trade names, commercial products, or organizations does not imply endorsement by the US Government.

## Supporting information


Data S1

Data S2

Data S3

## Data Availability

Data sharing is not applicable to this article as no new data were created or analyzed in this study.
