# Supplementary material for: Behavioral interventions for individuals with fetal alcohol spectrum disorder: A review of systematic reviews
Source: Alcohol Clin Exp Res (Hoboken). 2025 Aug 11;49(9):2064–75. doi: 10.1111/acer.70129 (PMC12463752; doi:10.1111/acer.70129)
Supplement: Supplementary file 1 — Data S1 [file ACER-49-2064-s002.docx]

**Supplementary Material 1. Search Strategy**

**PubMed**

("Fetal Alcohol Spectrum Disorders"[Mesh] OR "Fetal Alcohol Spectrum Disorder*"[tiab] OR "Fetal Alcohol Syndrome"[tiab] OR “Partial Fetal Alcohol Syndrome”[tiab] OR “Alcohol-Related Birth Defects”[tiab] OR “Prenatal Alcohol Exposure”[tiab] OR "Alcohol-Related Neurodevelopmental Disorder"[tiab] OR “Neurobehavioral Disorder Associated With Prenatal Alcohol Exposure”[tiab])

**AND**

(“Behavior*” OR “Intervention*”[tiab] OR “Behavioral Intervention*” OR “Behavior Therapy”[Mesh] OR “Behavioral Therapy” OR “Behavioral Treatment”OR “Behavioral Management” OR “Behavioral Support” OR “Behavioral Counseling” OR “Self-Control”[Mesh] OR “Emotional Regulation”[Mesh] OR “Self-Regulation” OR “Self Regulation” OR “Adaptive Behavior” OR “Maladaptive Behavior*”)

**AND**

(“Infant”[Mesh] OR “Child, Preschool”[Mesh] OR “Child”[Mesh] OR  “Adolescent”[Mesh] OR Infant* OR Child* OR Children OR Youth OR Adolescent* OR Teenager OR Pediatric*)

**AND**

(“Systematic Review”[tiab] OR “Review”[tiab])

**AND**

(2005:2024[pdat])

**APA PsycInfo**

(TI “Fetal Alcohol Spectrum Disorder*” OR AB “Fetal Alcohol Spectrum Disorder*” OR TI "Fetal Alcohol Syndrome" OR AB "Fetal Alcohol Syndrome" OR TI “Prenatal Alcohol Exposure” OR AB “Prenatal Alcohol Exposure” OR TI “Prenatal Exposure” OR AB “Prenatal Exposure” OR TI “Partial Fetal Alcohol Syndrome” OR AB “Partial Fetal Alcohol Syndrome” OR TI  “Alcohol-Related Birth Defects” OR AB “Alcohol-Related Birth Defects” OR TI “Alcohol-Related Neurodevelopmental Disorder” OR AB “Alcohol-Related Neurodevelopmental Disorder” OR TI  “Neurobehavioral Disorder Associated With Prenatal Alcohol Exposure” OR AB “Neurobehavioral Disorder Associated With Prenatal Alcohol Exposure”)

AND

(Behavior* OR Intervention* OR “Behavioral Intervention*” OR “Behavior Therapy” OR “Behavioral Therapy” OR “Behavioral Treatment” OR “Behavioral Management” OR “Behavioral Support” OR “Behavioral Counseling” OR “Behavior Problems” OR “Behavior Modification” OR “Behavior Change” OR “Self-Control” OR “Self-Regulation” OR “Self Regulation” OR “Emotional Regulation” OR “Adaptive Behavior” OR “Maladaptive Behavior*”)

AND

(Infant* OR Child OR Children OR Adolescent* OR Youth OR Teenager OR Pediatric*)

AND

(TI “Systematic Review*” OR AB “Systematic Review*” OR TI Review OR AB Review)

Filter: 2005-2024

**Embase**

(‘Fetal Alcohol Spectrum Disorder*’/exp OR 'Fetal Alcohol Spectrum Disorder*':ti,ab OR ‘Fetal Alcohol Syndrome’:ti,ab OR ‘Partial Fetal Alcohol Syndrome’:ti,ab OR ‘Alcohol-Related Birth Defects’:ti,ab OR ‘Prenatal Alcohol Exposure’:ti,ab OR ‘Alcohol-Related Neurodevelopmental Disorder’:ti,ab OR ‘Neurobehavioral Disorder Associated With Prenatal Alcohol Exposure’:ti,ab)

 AND

(Behavior* OR Intervention*:ti,ab OR ‘Behavioral Intervention*’ OR ‘Behavior Therapy’ OR ‘Behavioral Therapy’ OR ‘Behavioral Treatment’ OR ‘Behavioral Management’ OR ‘Behavioral Support’ OR ‘Behavioral Counseling’ OR ‘Self-Control’ OR ‘Self Control’/exp OR ‘Emotional Regulation’ OR “Self-Regulation” OR “Self Regulation” OR ‘Adaptive Behavior’ OR ‘Maladaptive Behavior’)

AND

 (Infant* OR Child* OR Children OR Adolescent* OR Youth OR Teenager OR Pediatric*)

AND

(‘Systematic Review’:ti,ab OR ‘Review’:ti,ab)

AND

[2005-2024]/py

**Web of Science**

(((((((((((((TI=("Fetal Alcohol Spectrum Disorder*")) OR TI=("Fetal Alcohol Syndrome")) OR TI=(“Partial Fetal Alcohol Syndrome”)) OR TI=(“Alcohol-Related Birth Defects”)) OR TI=(“Prenatal Alcohol Exposure”)) OR TI=("Alcohol-Related Neurodevelopmental Disorder")) OR TI=(“Neurobehavioral Disorder Associated With Prenatal Alcohol Exposure”)) OR AB=("Fetal Alcohol Spectrum Disorder*")) OR AB=("Fetal Alcohol Syndrome")) OR AB=(“Partial Fetal Alcohol Syndrome”)) OR AB=(“Alcohol-Related Birth Defects”)) OR AB=(“Prenatal Alcohol Exposure”)) OR AB=("Alcohol-Related Neurodevelopmental Disorder")) OR AB=(“Neurobehavioral Disorder Associated With Prenatal Alcohol Exposure”)

AND

(((((((((((((((ALL=(Behavior*)) OR TI=(Intervention*)) OR AB=(Intervention*)) OR ALL=(“Behavioral Intervention*” )) OR ALL=(“Behavior Therapy” )) OR ALL=(“Behavioral Therapy” )) OR ALL=(“Behavioral Treatment” )) OR ALL=(“Behavioral Management” )) OR ALL=(“Behavioral Support” )) OR ALL=(“Behavioral Counseling” )) OR ALL=(“Self-Control” )) OR ALL=(“Emotional Regulation”)) OR ALL=(“Self-Regulation”)) OR ALL=(“Self Regulation”)) OR ALL=(“Adaptive Behavior” )) OR ALL=(“Maladaptive Behavior*”)

AND

((((((ALL=( Infant* )) OR ALL=(Child*)) OR ALL=(Children)) OR ALL=(Adolescent*)) OR ALL=(Youth )) OR ALL=(Teenager)) OR ALL=(Pediatric*)

AND

(((TI=(“Systematic Review”)) OR TI=(Review)) OR AB=(“Systematic Review”)) OR AB=(Review)

Filter: 2005-2024

**Medline**

1. exp Fetal Alcohol Spectrum Disorders/

2. "Fetal Alcohol Spectrum Disorder*".ti. or "Fetal Alcohol Spectrum Disorder*".ab. or "Fetal Alcohol Syndrome".ti. or "Fetal Alcohol Syndrome".ab. or "Partial Fetal Alcohol Syndrome".ti. or "Partial Fetal Alcohol Syndrome".ab. or "Alcohol-Related Birth Defects".ti. or "Alcohol-Related Birth Defects".ab. or "Prenatal Alcohol Exposure".ti. or "Prenatal Alcohol Exposure".ab. or "Alcohol-Related Neurodevelopmental Disorder".ti. or "Alcohol-Related Neurodevelopmental Disorder".ab. or "Neurobehavioral Disorder Associated With Prenatal Alcohol Exposure".ti. or "Neurobehavioral Disorder Associated With Prenatal Alcohol Exposure".ab.

3. Behavior*.af. or Intervention.ti. or Intervention.ab. or "Behavioral Intervention*".af. or "Behavioral Therapy".af. or "Behavioral Treatment".af. or "Behavioral Management".af. or "Behavioral Support".af. or "Behavioral Counseling".af. or "Self-Regulation".af. or "Self Regulation".af. or "Adaptive Behavior".af. or "Maladaptive Behavior*".af.

4. exp Behavior Therapy/

5. exp Self-Control/

6. exp Emotional Regulation/

7. (Infant* or Child* or Children or Youth or Adolescent or Teenager or Pediatric).af.

8. Systematic Review.ti. or Systematic Review.ab. or Review.ti. or Review.ab.

9. 1 or 2

10. 3 or 4 or 5 or 6

11. 7 and 8 and 9 and 10

Filter: 2005-2024
