# Supplementary material for: Behavioral interventions for individuals with fetal alcohol spectrum disorder: A review of systematic reviews
Source: Alcohol Clin Exp Res (Hoboken). 2025 Aug 11;49(9):2064–75. doi: 10.1111/acer.70129 (PMC12463752; doi:10.1111/acer.70129)
Supplement: Supplementary file 2 — Data S2 [file ACER-49-2064-s001.docx]

| **Supplemental Material 2.** Characteristics and findings of the included systematic reviews | | | | | | | | |
| --- | --- | --- | --- | --- | --- | --- | --- | --- |
| **Author (Year)** | **Type of Review** | **Proportion of Relevant Studies^a^** | **Years of Relevant Studies** | **Study Designs (Number of Studies)** | **Description of Behavioral Intervention(s)** | **Population Characteristics** | **Behavioral Intervention Settings (Number of Studies)** | **Key Findings of Behavioral Studies** |
| Hilly et al., 2023 | Systematic Review & Meta-Analysis | 11/25 | 2006-2021 | - Randomized control trials (RCTs) (5) - Case-control trials (CCTs) (4) - Single case experimental designs (SCEDs) (2) | - **GoFAR (3-arm RCT):** Computer-based game to teach metacognitive control;   **Faceland:** Computer-based game to teach emotion recognition;  **Behavior Analog Therapy:** Application of FAR strategy for daily living skills.   - **Alert Program for Self Regulation (CCT):** Manualized intervention for self-regulation using a car engine analogy. - **Neurocognitive Habilitation Therapy (RCT):** Adaptation of Alert Program, TBI-related strategies, and psychoeducation. - **Children’s Friendship Training (CCT):** Group training to improve social skills and reduce problem behaviors. - **Self-management intervention (SCED):** Behavioral-monitoring intervention involving independent homework and bedroom cleaning tasks. | - Countries: USA, Canada - N = 385 - Age range: 5-12 years - Diagnoses^*^: FAS, pFAS, ARND - Ethnicities (Avg): 30.6% Caucasian; 7.8% African American;  0.4% Indigenous American;  4.7 % Hispanic; 0.52% Asian;  7.4 % Mixed/Other; 23.4% No Response/Missing | - Clinic Setting (8) - Home (2) - Community Center (1) | - One of three studies assessing GoFar found significant reductions in disruptive behaviors for both GoFAR and Faceland groups. - Three studies using Alert and/or Neurocognitive Habilitation Therapy found consistent improvements in emotional regulation and social-problem solving for the treatment groups. - Three studies using Children’s Friendship Training found inconsistent improvements in social skills knowledge and reduced problem behaviors for the treatment groups. - Two case studies assessing a self-management intervention found reductions in intensity of problem behaviors, which were not sustained with intervention withdrawal. - Sub-group analysis found statistically significant treatment effect for behavioral outcomes, however, certainty in the evidence was rated low due to concerns of high risk of bias from caregiver ratings and small sample sizes. |
| Flannigan et al., 2020 | Systematic Review | 15/33 | 2006-2019 | - Randomized control trials (RCTs) (8) - Case-control trials (CCTs) (4) - Single case experimental designs (SCEDs) (3) | - **GoFAR, Alert Program for Self Regulation, Neurocognitive Habilitation Therapy, Children’s Friendship Training (CFT), Self-management intervention:** See Hilly et al., 2023 - **Families on Track (RCT):**  Teaches skills to improve social competence and decrease behavioral problems in children whiles simultaneously assisting with family-level factors. - **Parents Under Pressure (SCED):** Therapy sessions to enhance self-regulation skills through improvement of child-caregiver relationship and teaching of mindfulness strategies. - **Parent-Child Interaction Therapy (RCT):** Child-caregiver educational and training sessions to improve child behavior and social skills. - **Applied-Behavior Analysis (ABA)-based Verbal Behavior Intervention (SCED):** Therapy sessions to improve adaptive and emotional behavior and functional communication skills | - Countries: USA, Canada, Australia - N = 460 - Age range: 3-12 years - Diagnoses^*^: FAS, pFAS, ARND, ND-PAE - Ethnicity: None Reported | - Clinic Setting (8) - Home (2) - Community Center (3) - Therapeutic Setting-Unspecified (2) | - For the findings of GoFAR, Alert, Neurocognitive Habilitation, CFT, and the self-management intervention, see Hilly et al., 2023. - Two studies assessing the effects of Families on Track found significant improvements in emotional regulation for the treatment group, which were not maintained at follow-up. Both treatment and control groups showed improvements in disruptive behavior and negative affect, which were maintained at follow-up. - A study evaluating the Parents Under Pressure program found that only one of two children improved with executive functioning behaviors, while both children improved in psychosocial functioning. - In a study of Parent-Child Interaction Therapy, children in both the treatment and control groups showed significant improvements in number and intensity of behavioral problems. - A case study of an ABA-based Verbal Behavior Intervention reported significant improvements in functional communication, adaptive behavior, and emotional functioning which were maintained at program discharge. - Overall, the review reported that the strongest evidence-based interventions involved self-regulation and social skills strategies. - Reported limitations included issues with selection bias and blinding, lack of long-term follow-up, small sample sizes, and the use of informant reporting (i.e., caregiver ratings) rather than direct measurement of functioning. |
| Betts et al., 2022 | Systematic Review and Meta-Analysis | 10/21 | 2012-2017 | - Randomized control trials (RCTs) (8) - Case-control trials (CCTs) (1) - Single case experimental designs (SCEDs) (1) | - **GoFAR, Alert Program for Self Regulation, Neurocognitive Habilitation Therapy, Families on Track, Parents Under Pressure:** See Hilly et al., 2023 and Flannigan et al., 2020 | - Countries: USA, Canada, Australia - N = 227 - Age range: 4-12 years - Diagnoses^*^: FAS, pFAS, ARND, ND-PAE - Ethnicities (Avg): 30.6% Caucasian; 7.8% African American;  0.4% Indigenous American;  4.7 % Hispanic; 0.52% Asian;  7.4 % Mixed/Other; | - Clinic Setting (7) - Home (1) - Community Center (2) | - For the findings of each behavioral intervention study, see Hilly et al. 2023 and Flannigan et al., 2020 - Three indirect measures of executive functioning that relate to behavior are discussed in the review: Behavioral regulation, emotional control, and inhibition. - Sub-group analyses of RCTs and quasi-experimental studies showed no statistically significant difference between treatment and control groups on behavioral regulation, emotional control, and inhibition measures. However, the effect estimates were derived from meta-analysis of small groups of studies (i.e. 2-3 per analysis) and should be interpreted cautiously. - Reported an overall lack of high-quality research studies on interventions for children with FASD. - Reported limitations included: High to serious risk of bias for all studies, limited ability to assess long-term impact of interventions due to lack of follow-up data, and the applicability and analytical power of evidence being limited by small sample sizes. |
| Champagne et al., 2023 | Scoping Review | 4/5 | 2015-2019 | - Randomized control trials (RCTs) (4) | - **GoFAR, Alert Program for Self-Regulation, Families on Track:** See Hilly et al., 2023 and Flannigan et al., 2020 | - Countries: USA, Canada - N= 85 - Age Range: 4-12 years - Diagnoses*: FAS, pFAS,, ARND - Demographics: None Reported | - Clinic Setting (4) | - Reported a scarcity of psychosocial interventions designed to address behavioral challenges, especially aggression, in children and adolescents with FASD. All studies focused on related aspects such as disruptive behavior and emotional regulation. - The majority of the included studies focus on child outcomes, rather than outcomes for the family as a whole. The authors identify this as an area of concern given the effects of externalizing behaviors on the well-being of caregivers and families of children with FASD. - Reported that all studies took place in-person at university or pediatric clinics which may limit the generalizability to other contexts and accessibility for families requiring online/telehealth interventions. - GoFAR and Families on Track have since been adapted for remote delivery, however, the effectiveness of these online interventions for reducing externalizing behaviors in children with FASD remains unknown. |
| Ordenewitz et al., 2021 | Systematic Review | 10/25 | 2006-2018 | - Randomized control trials (RCTs) (6) - Case-control trials (CCTs) (4) | - **GoFAR, Alert Program for Self Regulation, Neurocognitive Habilitation Therapy, Families on Track, Children’s Friendship Training:** See Hilly et al., 2023 and Flannigan et al., 2020 | - Countries: USA, Canada - N = 413 - Age range: 4-12 years - Diagnoses^*^: FAS, pFAS, ARND - Demographics: None Reported | - Clinic Setting (8) - Community Center (2) | - For the findings of each behavioral intervention study, see Hilly et al. 2023 and Flannigan et al., 2020. - Reported that there were very few RCTS on interventions for children and adolescents with FASD, however, the available research to support therapeutic interventions in this population are promising. - Interventions that involve the family (e.g. therapy and educational sessions for both children with FASD and their caregivers) seem to be the most effective treatment approaches. Interventions relying on parent training only are less effective in improving child behavior. - Interventions involving teaching of self-regulation and social interaction skills appear to have transferable impact on attention and behavior. - Providing behavioral interventions through FASD experienced clinics and/or community-based mental health centers seem to be very beneficial for affected families. - Nearly all behavioral interventions studies were performed in younger children, highlighting the need for more research on the effectiveness of these interventions in adolescents. - Reported the limitation of potentially biased results due to caregiver reporting of child outcomes. |
| Reid et al., 2015 | Systematic Review | 7/32 | 2006-2015 | - Randomized control trials (RCTs) (3) - Case-control trials (CCTs) (4) | - **Alert Program for Self Regulation, Neurocognitive Habilitation Therapy, Children’s Friendship Training (CFT), and Parent-Child Interaction Therapy (PCIT):** See Hilly et al., 2023 | - Countries: USA, Canada - N = 399 - Age range: 3-12 years - Diagnoses^*^: FAS, pFAS, ARND - Demographics: None Reported | - Clinic Setting (5) - Community Center (1) - Therapeutic Setting-Unspecified (1) | - For the findings of GoFAR, Alert, Neurocognitive Habilitation, CFT, and PCIT, see Hilly et al., 2023 and Flannigan et al., 2020. - The identified interventions demonstrated potential for enhancing functioning across multiple domains. - Studies focusing on enhancing self-regulation and attentional control in early to middle childhood provided substantial evidence for behavioral and neuropsychological gains. However, these studies had little to no follow-up, which limited the ability to assess the long-term impact of the interventions. - Studies involving social skills interventions were methodologically strong and showed promising results. - Noted the need to explore more interventions for infants, young children, and adolescents. - Proposed that interventions may benefit from a more ecological stance rather than domain-specific focus, considering the complexity of circumstances affecting individuals with FASD. - Identified multiple study limitations including weak to moderate ratings on selection bias and blinding, small sample sizes and limited follow-up. |
| Peadon et al., 2009 | Systematic Review | 1/12 | 2006 | - Case-control trial (1) | - **Children’s Friendship Training:** See Hilly et al., 2023 | - Country: USA - N = 100 - Age range: 6-12 years - Diagnoses^*^: FAS, pFAS, ARND - Demographics: None Reported | - Clinic Setting (1) | - Found limited amount of studies addressing interventions for children with FASD. - Only one study related to behavior took place outside of a school setting: Children’s Friendship Training (CFT). - Reported that CFT only showed improvements in parent-reported social skills and reduced behavioral problems, however, teacher ratings showed no significant improvement in children’s behavior. The parent reported gains were maintained at three months follow-up. - Identified strength in the use of standardized outcome measures for the intervention studies. - Reported limitations included: Short term follow-up, unclear blinding, and potential selection bias. - Identified seven intervention studies for children with FASD that were in progress. |

a. Number of Relevant Behavioral Intervention Studies / Total Number of Studies in the Review; *FAS – Fetal Alcohol Syndrome, pFAS – Partial Fetal Alcohol Syndrome, ARND – Alcohol-Related Neurodevelopmental Disorder, ND-PAE – Neurobehavioral Disorder Associated With Prenatal Alcohol Exposure
