# Supplementary material for: Behavioral interventions for individuals with fetal alcohol spectrum disorder: A review of systematic reviews
Source: Alcohol Clin Exp Res (Hoboken). 2025 Aug 11;49(9):2064–75. doi: 10.1111/acer.70129 (PMC12463752; doi:10.1111/acer.70129)
Supplement: Supplementary file 3 — Data S3 [file ACER-49-2064-s003.docx]

# Supplemental Material 3. Excluded Studies Table

| Full Reference | Type | Summary | Exclusion Reason |
| --- | --- | --- | --- |
| Paley, B., & O’Connor, M. J. (n.d.). Behavioral Interventions for Children and Adolescents with Fetal Alcohol Spectrum Disorders. Alcohol Research & Health, 64–75. | Review | Reviews behavioral interventions for children with FASD, emphasizing direct child-focused strategies; limited coverage of parent-only interventions. | Not a systematic review |
| Tan-MacNeill, K. M., Smith, I. M., Johnson, S. A., Chorney, J., & Corkum, P. (2021). A systematic review of online parent-implemented interventions for children with neurodevelopmental disorders. Children's Health Care, 50(3), 239–277. https://doi.org/10.1080/02739615.2021.1886934 | Systematic Review | Evaluates online parent-implemented interventions; most target child outcomes indirectly through parent behavior change; quality of evidence generally low. | Behavioral interventions only targeted caregivers |
| Premji, S., Benzies, K., Serrett, K., & Hayden, K. A. (2006). Research-based interventions for children and youth with a Fetal Alcohol Spectrum Disorder: revealing the gap. Child: Care, Health and Development, 33(4), 389–397. | Systematic Review | Identifies limited experimental research on interventions for FASD; emphasizes lack of robust studies on parent-only approaches. | Wrong targeted intervention |
| Dixon, D. R., Kurtz, P. F., & Chin, M. D. (2008). A systematic review of challenging behaviors in children exposed prenatally to substances of abuse. Research in Developmental Disabilities, 29(6), 483–502. | Systematic Review | Highlights challenging behaviors in substance-exposed children; notes few studies on intervention, especially those targeting parents. | Not focused on interventions |
| Varigonda, A. L., Edgcomb, J. B., & Zima, B. T. (2020). The impact of exercise in improving executive function impairments among children and adolescents with ADHD, autism spectrum disorder, and fetal alcohol spectrum disorder: a systematic review and meta-analysis. Archives of Clinical Psychiatry, 47(5), 146–156. | Systematic Review and Meta-Analysis | Assesses effects of exercise on executive functioning in neurodevelopmental disorders; focuses on child-directed outcomes. | Wrong targeted intervention |
| Koren, G. (2015). Pharmacological Treatment of Disruptive Behavior in Children with Fetal Alcohol Spectrum Disorder. Pediatric Drugs, 17, 179–184. https://doi.org/10.1007/s40272-015-0118-4 | Review | Reviews pharmacological approaches for managing disruptive behavior in children with FASD; highlights lack of controlled studies, with a focus on stimulant and antipsychotic use. | Wrong targeted intervention |
| Khoury, J. E., Milligan, K., & Girard, T. A. (2015). Executive Functioning in Children and Adolescents Prenatally Exposed to Alcohol: A Meta-Analytic Review. Neuropsychology Review, 25(2), 149–170. https://doi.org/10.1007/s11065-015-9289-6 | Meta-Analysis | Meta-analyzes executive function deficits in youth with FASD; confirms significant impairments in working memory, inhibition, and set shifting. | Not focused on interventions |
| Leruste, S., et al. (2024). Scoping Review on the Role of the Family Doctor in the Prevention and Care of Patients with Foetal Alcohol Spectrum Disorder. BMC Primary Care, 25:66. https://doi.org/10.1186/s12875-024-02291-x | Scoping Review | Examines GP roles in prevention, diagnosis, and follow-up of FASD; primarily focused on healthcare system integration rather than intervention effectiveness. | Focused on frameworks for interventions |
| Rigney, G., et al. (2018). A Systematic Review to Explore the Feasibility of a Behavioural Sleep Intervention for Insomnia in Children with Neurodevelopmental Disorders. Sleep Medicine Reviews, 41, 244–254. https://doi.org/10.1016/j.smrv.2018.03.008 | Systematic Review | Explores behavioral sleep interventions for children with NDDs including FASD; supports feasibility of transdiagnostic approaches with parent training as a key component. | Wrong targeted intervention |
